# Imaging and Characterization of Oxidative Protein Modifications in Skin

**DOI:** 10.3390/ijms24043981

**Published:** 2023-02-16

**Authors:** Ankush Prasad, Hana Duchová, Renuka Ramalingam Manoharan, Deepak Rathi, Pavel Pospíšil

**Affiliations:** Department of Biophysics, Faculty of Science, Palacký University, Šlechtitelů 27, 783 71 Olomouc, Czech Republic

**Keywords:** porcine skin, protein modification, oxidative radical reaction, protein carbonyls, two-dimensional imaging, ultra-weak photon emission, malondialdehyde, reactive oxygen species

## Abstract

Skin plays an important role in protection, metabolism, thermoregulation, sensation, and excretion whilst being consistently exposed to environmental aggression, including biotic and abiotic stresses. During the generation of oxidative stress in the skin, the epidermal and dermal cells are generally regarded as the most affected regions. The participation of reactive oxygen species (ROS) as a result of environmental fluctuations has been experimentally proven by several researchers and is well known to contribute to ultra-weak photon emission via the oxidation of biomolecules (lipids, proteins, and nucleic acids). More recently, ultra-weak photon emission detection techniques have been introduced to investigate the conditions of oxidative stress in various living systems in in vivo, ex vivo and in vitro studies. Research into two-dimensional photon imaging is drawing growing attention because of its application as a non-invasive tool. We monitored spontaneous and stress-induced ultra-weak photon emission under the exogenous application of a Fenton reagent. The results showed a marked difference in the ultra-weak photon emission. Overall, these results suggest that triplet carbonyl (^3^C=O^∗^) and singlet oxygen (^1^O_2_) are the final emitters. Furthermore, the formation of oxidatively modified protein adducts and protein carbonyl formation upon treatment with hydrogen peroxide (H_2_O_2_) were observed using an immunoblotting assay. The results from this study broaden our understanding of the mechanism of the generation of ROS in skin layers and the formation/contribution of various excited species can be used as tools to determine the physiological state of the organism.

## 1. Introduction

Numerous crucial bodily processes and functions, such as thermoregulation, metabolism, sensory perception, excretion, hormones, and vitamin synthesis, can be attributed to the largest organ, the skin, either partially or to their full extent [1]. Additionally, the skin represents the primary mechanical obstruction that secures the body against invading pathogens, solar irradiation, fluctuating temperature, dehydration, and various chemical and mechanical aggressions [2,3,4]. Therefore, it is imperative to study the mechanisms of its multitudinous role to discover novel effective treatments/procedures to maintain/restore its healthy condition. To achieve this, researchers often turn to animal models as a representation of human skin [5,6]. In addition to alleviating ethical and financial burdens, the variety of model systems and tools available makes them an attractive alternative [7,8,9]. These systems must possess high levels of similarity with human skin in terms of the attribute(s) that are integral to a given research purpose. Pig/porcine skin ranks at the top (particularly in dermatology) due to the myriad anatomical, biochemical, and physiological similarities found in pigs and humans [10]. Its advantage also lies in its widespread accessibility and cost-effectiveness, which can be traced to pigs being the most produced/consumed meat worldwide [11]. Similarities are found starting from the multilayered skin composition, which can be grouped into three main layers: the epidermis, the dermis, and the hypodermis (also called subcutis) [10,12]. However, no animal model system shares all the features of human skin in its entirety. For our study, porcine skin was selected as an ex vivo model, owning to its comparably higher similarity with human skin in the features essential for our research (such as its morphology and biochemical composition) than the other available models.

Skin is rich in reactive oxygen species (ROS), such as, but not limited to, hydrogen peroxide (H_2_O_2_) and hydroxyl radical (HO^•^) [7,13,14]. This is due to their endogenous and exogenous production/stimulation. Constant contact with molecular oxygen (O_2_), xenobiotics, solar radiation, oxidative metabolism, pathogens destroying immune cells, physiological and psychological stress are examples that affect the delicate balance of natural oxidants/antioxidants in the cell and, subsequently, the tissue/organ/organism [15,16]. Under regulated conditions, ROS are generally neutralized by a network of non-enzymatic antioxidants, such as glutathione and ascorbic acid or enzymatic antioxidants such as superoxide dismutase (SOD), catalase (CAT), glutathione peroxidase (GPX), glutathione reductase, and thioredoxin reductase (TRX). A key enzyme that detoxifies H_2_O_2_ is the peroxisomal localized catalase. Enzymatic antioxidants act in a coordinated way to maintain normal redox homeostasis [13,17]. If this condition is not restored through innate enzymatic and non-enzymatic mechanisms/exogenous antioxidants, it leads to a condition called oxidative stress [18,19]. The eustress is attributed to ROS involved in cellular signaling, but the risk comes when oxidative stress prevails, reaches toxic levels, and important biomolecules are negatively affected [18]. Biomolecule oxidation was found to be the cause/factor in the formation/aggravation of several diseases, such as diabetes, psoriasis, Alzheimer’s disease, and other diseases related to age [20,21]. Oxidative stress-induced protein modifications are a common feature in several pathologies and are routinely employed as a marker of oxidative processes, along with malondialdehyde (MDA), which is a by-product of lipid peroxidation [22].

This study aims to increase our understanding of ROS-induced oxidative stress in skin. Exogenous oxidant (H_2_O_2_), with or without transition metal ions, was used to mimic chemical/environmental pollutants. The exogenous use of transition metals enhances the oxidative process drastically; thus, its use in our two-dimensional studies was intended to enhance the subsequent photon emission, described later in the section. Using a non-invasive ultrasensitive charge-coupled device (CCD) camera, a ^1^O_2_ scavenger (sodium ascorbate), and the interference filter, we attempted to understand the degree of damage to biomolecules reflected by ultra-weak photon emission. As skin is rich in iron and other transition metals, we believe that the cascade of reactions (mediated by the formation of the HO^•^) might have played a role in the eventual oxidative damage to lipids and proteins. To further understand the mechanism and possible oxidative consequences, we used protein immunoblotting, where anti-MDA and anti-DNP antibodies were used to observe the protein modification.

## 2. Results and Discussion

### 2.1. Spontaneous and Fenton Reagent-Induced Ultra-Weak Photon Emission from Skin

**Figure 1:**
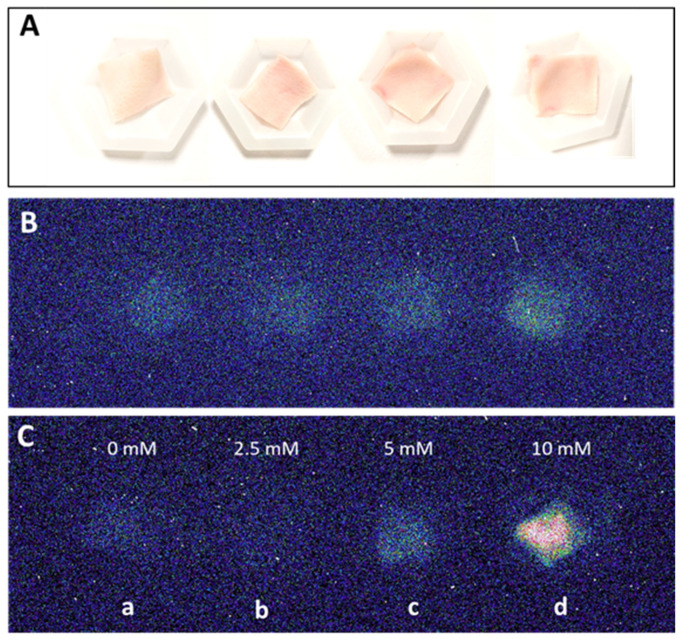
Two-dimensional photon emission imaging using a CCD camera from skin biopsies. Photographs (**A**) and spontaneous two-dimensional ultra-weak photon emission images (**B**). In (**C**), induced photon emission images were captured after topical treatment of skin biopsies with 2.5 mM, 5 mM and 10 mM (**b**–**d**) H_2_O_2_/250 µM FeSO_4_ versus control (**a**). Dark adaptation of 30 min was conducted prior to the exogenous application of FeSO_4_ and H_2_O_2_.

**Figure 2:**
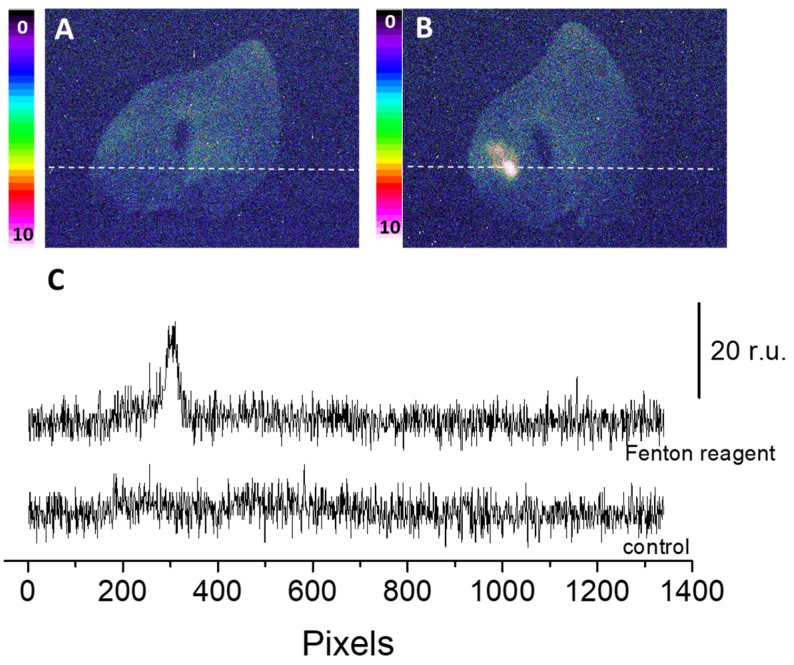
Spontaneous (**A**) and Fenton reagent-induced two-dimensional image of the ultraweak photon emission (**B**) from a porcine ear. In B, 10 mM H_2_O_2_/250 µM FeSO_4_ was topically applied. All other experimental conditions are as described in Figure 1. In (**C**), the Y-axis shows the number of photon counts after 30 min accumulation, whereas the X-axis denotes the pixel of the image.

The two-dimensional image of the ultra-weak photon emission was measured spontaneously from the porcine ears and after the topical application of the Fenton reagent in the setup shown and described in detail in Section 3. Variable concentrations of H_2_O_2_ (0, 2.5 mM, 5 mM, and 10 mM) and FeSO_4_ were topically applied to the skin biopsies and the corresponding ultra-weak photon emission images were captured (Figure 1). The upper panel (Figure 1A) shows the photographs of the prepared skin biopsies; Figure 1B shows the spontaneous ultra-weak photon emission images to demonstrate any variability in the spontaneous ultra-weak photon emission; Figure 1C shows the dependence of the ultra-weak photon emission with the increasing concentration of Fenton reagent. It is obvious that with the increasing concentration of oxidants, there is a corresponding increase in the intensity of the ultra-weak photon emission. Following the optimization, we further carried out our study on ex vivo porcine ear, where the treatment condition was limited to 10 mM H_2_O_2_/250 µM FeSO_4_ (Figure 2). Figure 2A (left panel) shows an image of the ultra-weak photon emission from an ex vivo porcine ear without any stimulation/induction of oxidative stress. In Figure 2B, the ultra-weak photon emission image was measured following the treatment with the Fenton reagent. Figure 2C shows the photon intensity at the pixels marked on the images by white dotted lines. As apparent from the intensity of the ultra-weak photon emission, the skin not treated with Fenton reagent (control) shows no enhancement, whereas the skin treated with Fenton reagent shows a maximum intensity of ~25 counts/pixel.

**Figure 3:**
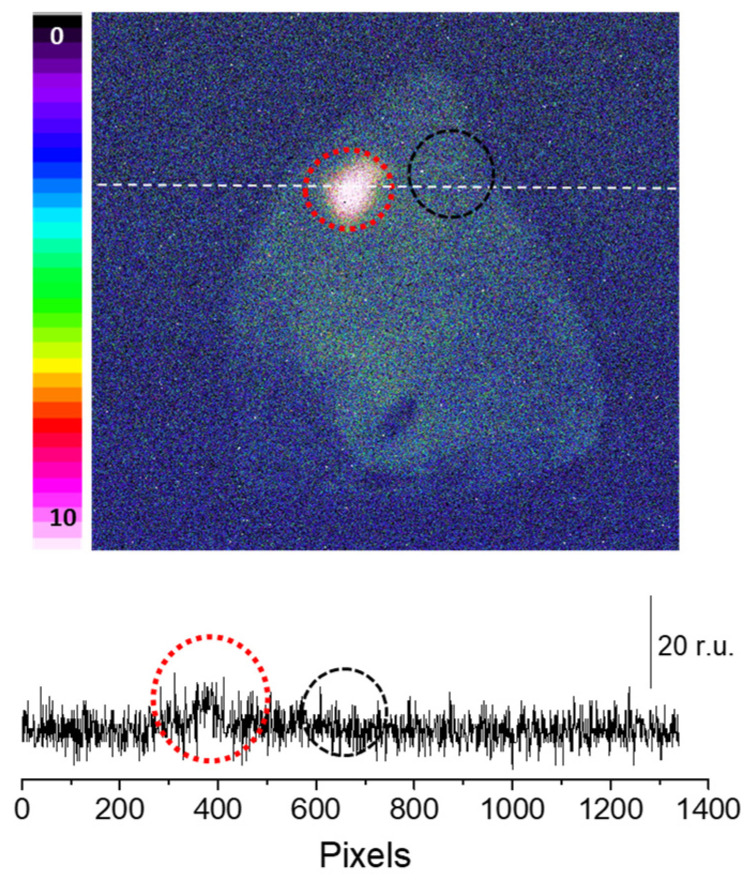
Fenton reagent-induced two-dimensional photon emission imaging from the porcine ear. The ultra-weak photon emission imaging was performed in a porcine ear treated with 10 mM H_2_O_2_/250 µM FeSO_4_ in the absence (red circle) and presence (black circle) of sodium ascorbate (10 mM). Samples were treated with sodium ascorbate 10 min before to the topical application of Fenton reagent. The Y-axis in the lower panel indicates the number of photon counts after 30 min of accumulation, whereas the X-axis shows the pixel of the image.

Sodium ascorbate (10mM), which is a scavenger of ^1^O_2_ [23], was topically applied to the porcine skin 10 min before the application of the Fenton reagent. It is evident that the presence of sodium ascorbate before the application of Fenton reagent noticeably lowered the ultra-weak photon emission (Figure 3). As evident from the intensity of the photon emission, the Fenton reagent-treated porcine skin shows a higher intensity, which was found to be suppressed almost completely in the case of the skin pretreated with sodium ascorbate. It is thus obvious that the involvement of ^1^O_2_ dimol photon emission in the overall ultra-weak photon emission can be substantial (Figure 3). The conclusion is based on the fact that in an oxygen-rich environment, the excitation energy from ^3^C=O^∗^ can be transferred to O_2_ via triplet-singlet energy transfer, which can lead to the formation of ^1^O_2_. The collision of two ^1^O_2_ results in photon emission in the red band of the spectrum (634 and 703 nm), referred to as dimol emission [24].

**Figure 4:**
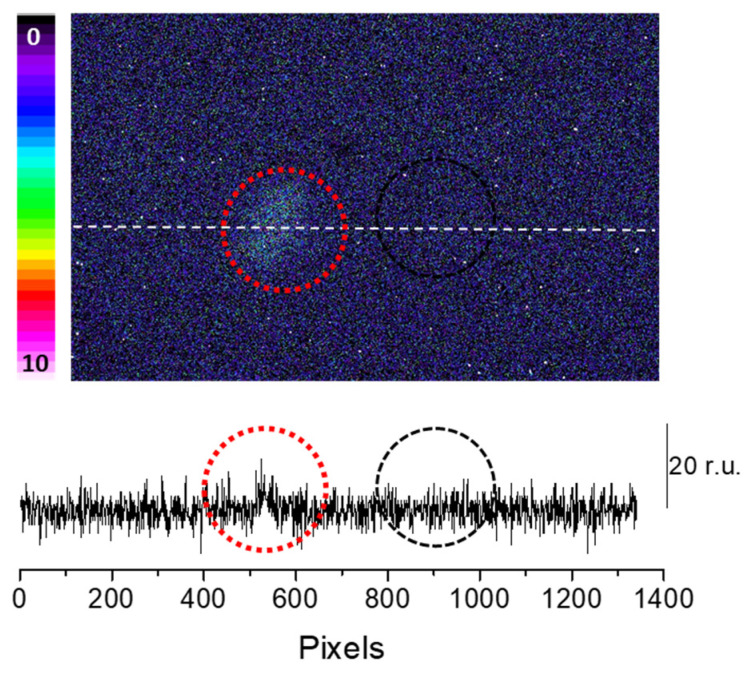
Fenton reagent-induced two-dimensional ultra-weak photon emission imaging from porcine ear measured after the topical application of Fenton reagent (10 mM H_2_O_2_/250 µM FeSO_4_) in the presence of interference filter type 644 (340–540 nm). The circle indicates the untreated (red) and treated (black) areas of the porcine skin with sodium ascorbate. Other experimental conditions as described in Figure 3. The Y-axis in the lower panel reflects the number of photon counts accumulated after 30 min, whereas the X-axis denotes the pixel of the image.

To confirm the claimed primary sources (^3^C=O^∗^) of the photon emission under the induced oxidative stress, we mounted a blue-green interference filter type 644 with a transparency between 340–540 nm in front of the objective lens with the experimental condition, as in Figure 3. It can be seen that if the transparency was limited in the range of the blue-green region, typically destined for ^3^C=O^∗^ emission, partial photon emission can still be captured (Figure 4). This indicates that ^3^C=O^∗^ can be one of the significant contributors of ultra-weak photon emission during oxidative radical reaction in the skin.

### 2.2. Protein Modification under Generated ROS

Reactive oxygen species create oxidative radical reactions in cells due to several cellular components, for example, DNA, protein, lipids, and carbohydrates undergo modifications [25]. In the present study, the proteins undergoing modification by ROS were characterized using an immunoblotting technique. We limited the stress induction to H_2_O_2_ treatment alone as such high oxidative damage is not necessarily required to study protein modification using western blotting; the level of endogenous transition metal ions is believed to be sufficient to mediate the process. On the contrary, to image photon emission as a result of oxidative damage, we need a moderate to high level of oxidative damage and, thus, metal ions were additionally supplemented.

**Figure 5:**
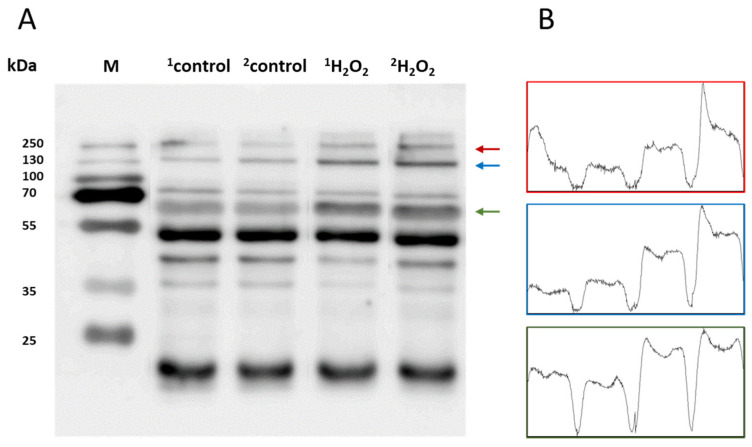
(**A**) Identification of MDA-protein adducts in homogenates of porcine skin cells. Lanes 1–2 show control samples, while lanes 3–4 are skin samples treated with H_2_O_2_ (10 mM) (**B**). Quantification of protein bands (by densitogram analysis) from an anti-MDA blot is presented, and proteins of interest are indicated by arrows.

**Figure 6:**
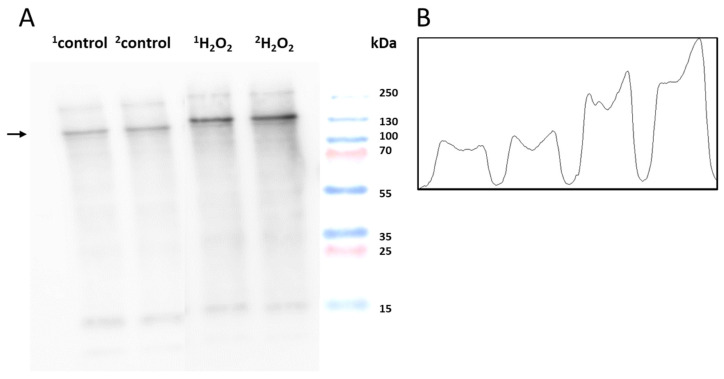
(**A**) Identification of DNP-carbonyl derivatives in homogenates of porcine skin cells. Lane 1–2 shows control sample, while lanes 3–4 are skin samples treated with H_2_O_2_ (10 mM) (**B**) Quantification of protein bands (by densitogram analysis) from an anti-DNP blot is presented, and the selected proteins of interest are indicated by arrows.

To study the protein modification (protein carboxylation and protein carbonyl formation), we used anti-MDA and anti-DNP, respectively. For characterization, skin biopsies treated with H_2_O_2_ (10 mM) and control non-treated samples were separated using SDS PAGE and the samples were loaded in duplicate. Anti-MDA antibodies bind to MDA-modified proteins, thereby enabling the detection of MDA-protein adducts. Malondialdehyde reacts specifically with amino acid residues such as Lys, Arg, His and Cys. With reference to the anti-MDA blot (Figure 5A), MDA-protein adduct formations were observed around 15 kDa, 45 kDa, 50 kDa, 65 kDa, 130 kDa and 250 kDa. However, the band density of 65 kDa, 130 kDa and 250 kDa proteins were found to be enhanced in comparison to the control untreated groups. Differences in the levels were represented as densitogram in separate panels for each protein (Figure 5B) and the mechanism involved is presented in Figure 6.

**Figure 7:**
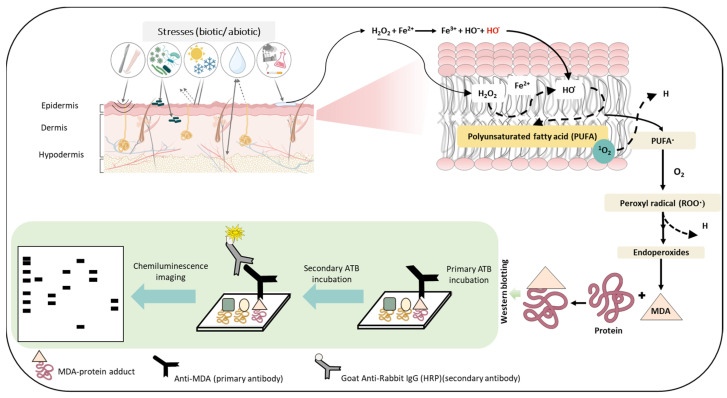
Schematic representation depicting the steps involved in the formation of the MDA-protein adduct as the consequence of ROS generation and successive oxidative radical reactions. The figures show stress factors (biotic and abiotic) that can lead to the formation of ROS which eventually can lead to lipid peroxidation and subsequently to the generation of the MDA-protein adduct. The lower panel depicts the use of immunoblotting techniques to detect MDA- protein formation.

**Figure 8:**
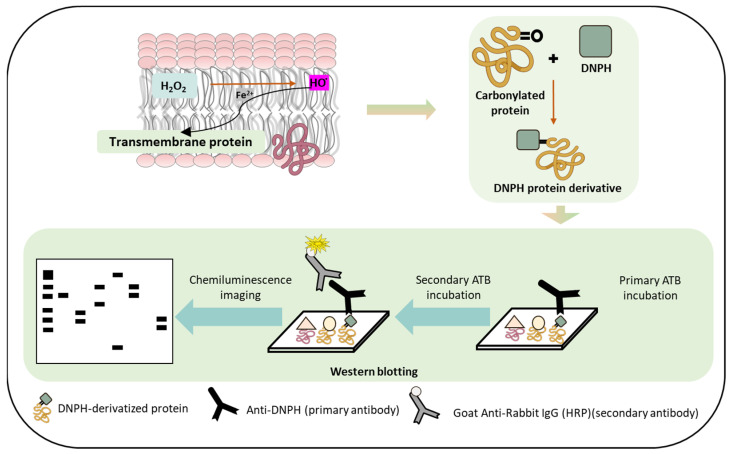
Schematic representation depicting the pathways of protein carbonyl formation which can occur as a result of protein oxidation. The lower panel depicts the use of immunoblotting techniques to detect protein carbonyl formation.

To monitor the protein carbonyl formation, derivatization was conducted, as mentioned in Section 3.4. The western blot analysis of the control and H_2_O_2_-treated skin biopsies displayed protein carbonyl levels, as measured by the anti-DNP antibodies (Figure 6A). A distinct band at 130 kDa was observed in both groups with varied patterns. It is clear that the carbonylated proteins isolated from the control groups are significantly less formed than the treatment groups. Figure 7 and Figure 8 (created with elements from BioRender.com) show the steps involved in the formation of the MDA-protein adduct and protein carbonyl formation, respectively. Differences between the control and treatment groups are presented as a separate densitogram (Figure 6B). Additional studies targeting the identification and characterization of selected proteins from both anti-MDA and anti-DNP blots are under study.

## 3. Materials and Methods

### 3.1. Porcine Skin

Porcine ears were obtained from a local slaughterhouse. They were transported at a low temperature (on ice) within the first 30 min. Whole ear/skin biopsies for two-dimensional imaging and immunoblotting were prepared according to the procedure described by Chiu and Burd (2005) [26], with minor modifications. Skin samples, collected each day, were used for each set of measurements.

### 3.2. Reagents and Antibodies

Fenton reagent preparation was conducted using H_2_O_2_ (Sigma-Aldrich Chemie GmbH, Mannheim, Germany) and ferrous sulfate (FeSO_4_.7H_2_O) (BDH Laboratory Supplies, Poole, UK). A variable concentration of H_2_O_2_ (2.5 mM, 5 mM, 10 mM) was used with a fixed concentration of iron sulphate (FeSO_4_) (250 µM) to chemically generate HO^•^. The procedure for topical application and its duration are specified in the figure legends, as applicable. Phosphatase and protease inhibitors were purchased from Roche (Mannheim, Germany). Rabbit polyclonal anti-MDA antibody was purchased from Abcam [anti-MDA antibody (ab27642)] (Cambridge, UK) and polyclonal goat anti-rabbit IgG conjugated with horseradish peroxidase (HRP) from Bio-Rad (Hercules, CA, USA). Rabbit polyclonal Dinitrophenyl-KLH antibody (anti-DNP) were procured from ThermoFisher scientific (Waltham, MA, USA).

### 3.3. Experimental Conditions and Setup for Two-Dimensional Imaging of Ultra-Weak Photon Emission

**Figure 9:**
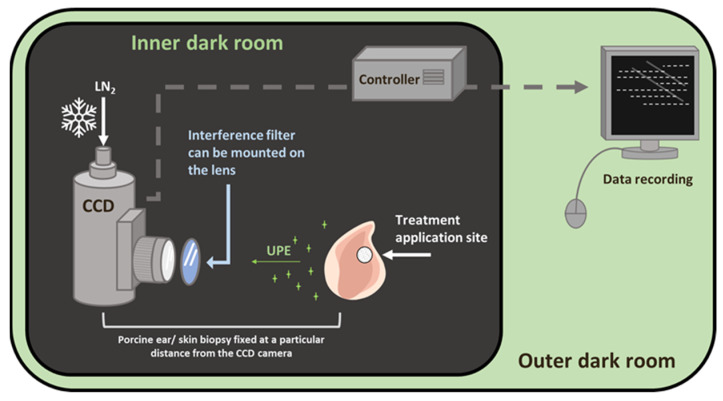
Schematic diagram of the experimental setup for two-dimensional photon emission imaging using a CCD camera. The diagram shows the inner dark room (gray) and the outer control room (green). The filter position for the spectral measurement was positioned in front of the objective lens, as shown.

A unique design of dark rooms is a prerequisite to avoid any interference by the absence of a photon. In the current study, all of the ultra-weak photon imaging measurements were conducted in an experimental dark room. Further details on the adopted methodology can be found in Prasad and Pospíšil (2013) [27]. The dark room, as well as the measurement setup, is shown in Figure 9. All of the experiments were carried out in three biological replicates, and the representative images have been presented. To study the spectral distribution of the ultra-weak photon emission in the oxidation reactions using Fenton reagents, filter type 644 (Schott and Gen, Jena, Germany), which is a blue-green interference filter with a transmission in the range 340–540 nm, was used and mounted in front of the objective lens of the CCD camera (Figure 1) [27].

Two-dimensional photon emission imaging was measured in porcine ear/skin biopsies utilizing a sensitive CCD camera. The skin samples were dark-adapted for 30 min to eradicate any interference by delayed luminescence and treated afterward. The other experimental conditions are as per the procedure described in the listed reference [27]. The VersArray 1300B CCD camera (Princeton Instruments, Trenton, NJ, USA) with a spectral sensitivity of 350–1000 nm and ~90% quantum efficiency was used under the following parameters: scan rate, 100 kHz; gain, 2; accumulation time, 30 min (porcine ear/skin biopsies). The CCD camera was cooled to −108 °C using a liquid nitrogen cooling system, which helps to reduce the dark current. Before each measurement, the data correction was made by subtracting the background noise from the experimental dataset.

### 3.4. Protein Immunoblotting

**Figure 10:**
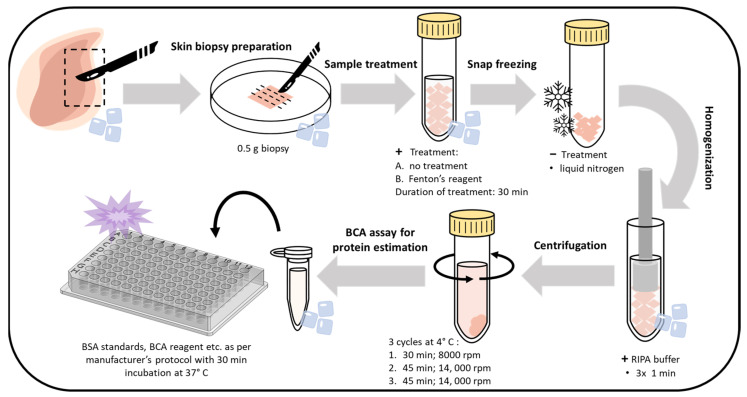
Steps showing the workflow and optimized protocol for whole-skin tissue lysate preparation, isolation, and the BCA assay for protein estimation.

Skin biopsies were prepared through initial washing with physiological solution (0.9% NaCl). First, 0.5 g of the skin biopsies were subjected to the desired treatment (control or Fenton reagent applied topically for 30 min each), followed by rinsing with distilled water. Subsequently, the samples were snap-freezed in liquid N_2_. The samples were then homogenized with radioimmunoprecipitation assay (RIPA) buffer (150 mM NaCl, 50 mM Tris (pH 8.0), 0.5% sodium deoxycholate, 0.1% SDS, and 1% NP-40) comprising 1% (*v*/*v*) protease and phosphatase inhibitor (*v*/*v*) (three times, 1 min each), followed by sequential centrifugations at 8000 rpm (30 min, 1 time) and 14,000 rpm (45 min, 2 times). The supernatant was collected and quantified with a Pierce BCA protein estimation kit (Thermo Fisher Scientific, Paisley, UK). The detailed sample preparation procedure is presented in Figure 10. Protein samples for Western blotting were prepared with SDS Laemmli sample buffer. The prepared samples were then subjected to electrophoresis and immunoblotting analysis using anti-MDA antibody. For immunoblotting, 2 biological replicates were performed for each measurement.

To detect protein carbonyl formation, the collected protein fractions were subjected to derivatization. Carbonyl groups present in the protein side chains were derivatized with 2,4 dinitrophenylhydrazine (DNPH), leading to the formation of stable 2,4 dinitrophenylhydrazone (DNP) derivative, which involves the addition of an equal volume of protein and 12% SDS (final concentration at 6%) and subsequent addition of 1X DNPH solution (50 mM solution in 50% sulphuric acid). The mixture was incubated at RT for 30 min and the reactions were neutralized with 2 M Tris base and 30% glycerol (0.75× *v*/*v* of DNPH solution). The resulting protein fractions were centrifuged at 14,000 rpm for 10 min and the supernatants were loaded onto SDS gels for immunoblotting with an anti-DNP antibody.

Whole cell homogenates (10 μg/lane), processed on 10% SDS gel, were then transferred to blotting membranes (nitrocellulose) using a Trans-Blot Turbo transfer system (Bio-Rad, Hercules, CA, USA). The membranes were blocked (BSA in phosphate buffered saline, pH 7.4, containing 0.1% Tween 20) overnight at 4 °C. The blocked membranes were probed for 2 h with an anti-MDA antibody at RT. After 4 cycles of washing with PBST and incubation for 1 h at room temperature with HRP-conjugated anti-rabbit secondary antibody (dilution 1:10,000) and subsequent washing [PBST, 5× (5 min each)], the immunocomplexes were visualized utilizing Immobilon Western Chemiluminescent HRP Substrate (Sigma Aldrich, GmbH, Mannheim, Germany) and imaged using an Amersham 600 imager (GE Healthcare, Amersham, UK). Densitometry analysis of the blots obtained was generated using Image J 1.53t [public domain software (Bethesda, MD, USA) provided by the National Institute of Mental Health, United States].

## 4. Conclusions

The skin is the primary interface between the body and environmental aggression and excessive production of reactive species, including ROS, and reactive nitrogen species have been known to form in skin tissues. Oxidative stress and its resulting oxidation products have been reported to be the main cause of ageing. Due to the limitation associated with the use of human skin for research purposes, porcine skin has been used as a model in the present study, with H_2_O_2_ as an exogenous oxidant. The two-dimensional spatiotemporal images and their spectral analysis confirmed the participation of triplet excited carbonyls and singlet oxygen dimol emission as substantial contributors resulting from oxidative radical reactions in the skin. The resultant oxidatively modified protein adducts and the differences in the band density of the selected proteins in the non-treated and H_2_O_2_ treated skin tissues were confirmed by blotting analysis. As oxidative stress remains a key factor in distinguishing physiological and pathological conditions, the present study helps to identify the specific protein targets involved in the process of oxidative damage in the skin. In addition, spatiotemporal imaging with a CCD camera, also demonstrated by Abdlaty and co-workers [4], is presented as a powerful tool for non-invasive imaging that has the potential to pave the way for its widespread usage in research and/or clinical trials.

## Data Availability

Generated data are presented in the manuscript and/or Supplementary Materials.

## Supplementary Materials

The following supporting information can be downloaded at: https://www.mdpi.com/article/10.3390/ijms24043981/s1.

## Abbreviations

ROS, reactive oxygen species; UPE, ultra-weak photon emission; RIPA, radio immunoprecipitation assay buffer; DNPH, dinitrophenylhydrazine; MDA, malondialdehyde.
