# Supplementary material for: Imaging and Characterization of Oxidative Protein Modifications in Skin
Source: Int J Mol Sci. 2023 Feb 16;24(4):3981. doi: 10.3390/ijms24043981 (PMC9959078; doi:10.3390/ijms24043981)
Supplement: Supplementary file 1 [file ijms-24-03981-s001.zip › ijms-2186671-supplementary.pdf]

Anti-MDA blot: Porcine samples

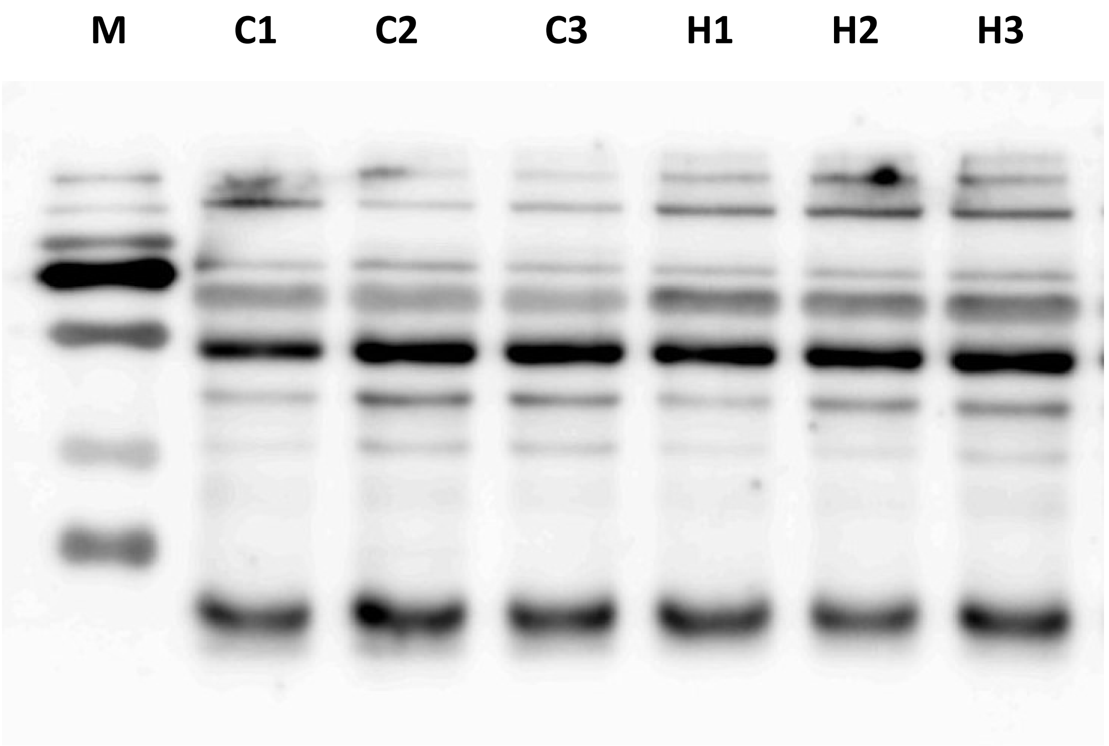

C1-C3: Control samples
H1-H3: H_2O_2 treated samples

Anti-DNPH blot: Porcine samples

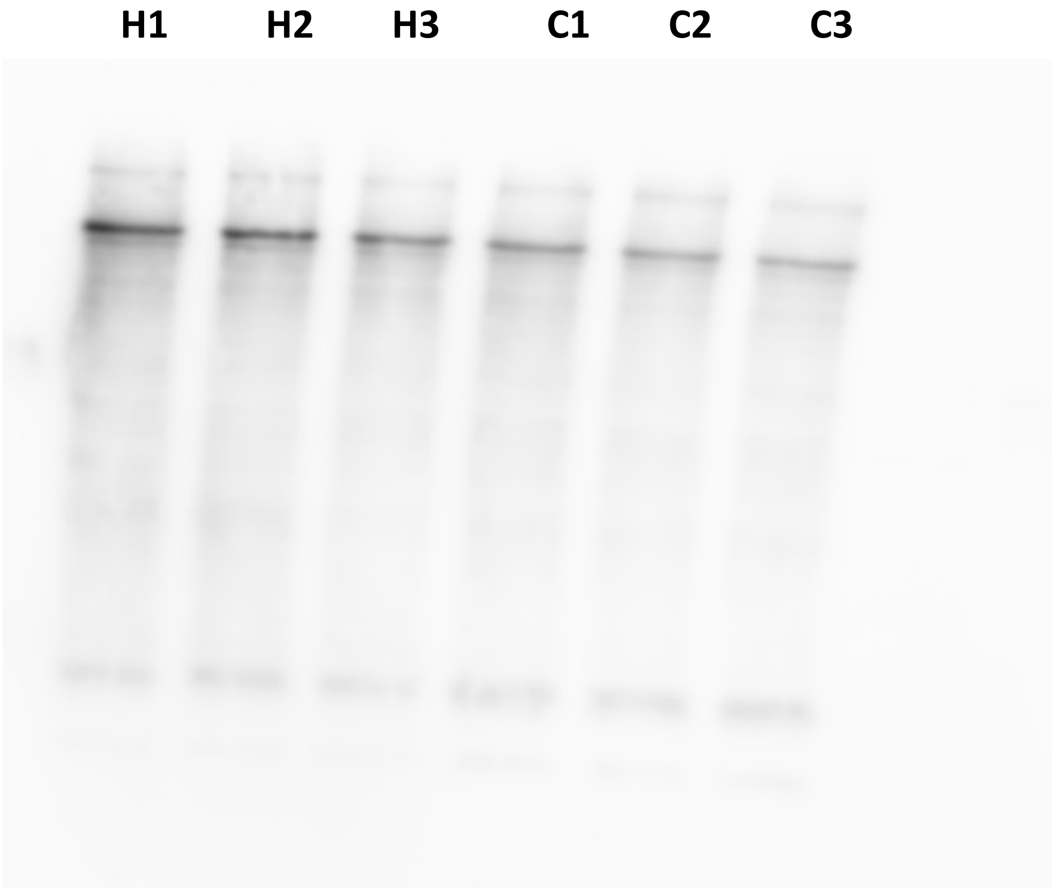

C1-C3: Control samples
H1-H3: H_2O_2 treated samples
